# Development of a Predictive Tool in Patients With High Pretest Probability for Transthyretin Amyloid Cardiomyopathy

**DOI:** 10.1016/j.jacadv.2026.102675

**Published:** 2026-03-25

**Authors:** Jocelyn Chai, Andrew Starovoytov, Daniel Worsley, Nowell M. Fine, Defen Peng, Sean Virani, Margot K. Davis

**Affiliations:** a Division of Cardiology, University of British Columbia, British Columbia, Canada; b Division of Radiology, University of British Columbia, British Columbia, Canada; c Division of Cardiology, University of Calgary, Alberta, Canada; d Centre for Cardiovascular Innovation, University of British Columbia, British Columbia, Canada

**Keywords:** cardiac amyloidosis, pyrophosphate, screening tool

## Abstract

**Background:**

Transthyretin amyloid cardiomyopathy (ATTR-CM) is an underdiagnosed cause of heart failure, arrhythmias, and valvular disease.

**Objectives:**

The objectives of the study were to develop: 1) screening criteria to identify high-risk patients for ATTR-CM; and 2) a predictive tool for ATTR-CM diagnosis.

**Methods:**

This prospective observational registry (2019-2022) at 2 Vancouver academic sites screened patients aged >60 years presenting to heart failure, atrial fibrillation, transcatheter valve clinics, and cardiologists’ offices. Patients meeting high-risk criteria who underwent technetium-99m-pyrophosphate were included. Predictors of ATTR-CM were identified using univariable and multivariable logistic regression.

**Results:**

Of 2,500 patients screened, 200 were enrolled (mean age 78.4 ± 8.3 years; 64.5% male) with a mean follow-up of 3 years. ATTR-CM was diagnosed in 46 (23.0%), and 7 (3.5%) had immunoglobulin light-chain amyloidosis amyloidosis. Compared to non–ATTR-CM, those with ATTR-CM were older (82.6 ± 7.1 years vs 77.2 ± 8.1 years; *P* < 0.001), male (80.4% vs 59.7%, *P* = 0.013), and had higher NT-proBNP (3,633 vs 2018; *P* = 0.027). ATTR-CM patients had greater posterior wall thickness (14.5 ± 3.2 vs 11.2 ± 1.8; *P* < 0.001), low QRS voltages (37.0% vs 3.9%; *P* < 0.001), more atrioventricular block (38.5% vs 19.2%; *P* = 0.018), and higher Doppler E/e’ (18.7 ± 7.6 vs 14.4 ± 6.2, *P* = 0.001). A 5-item predictive tool (score ≥7) identified patients who had positive ATTR-CM diagnoses (sensitivity 89.1% [95% CI: 80.1%-98.1%], specificity 85.1% [95% CI: 70.4%-90.7%], area under receiver operating characteristic curve 0.931 [95% CI: 0.884-0.978]).

**Conclusions:**

Broad screening criteria of high-risk populations referred for technetium-99m-pyrophosphate yielded new ATTR-CM diagnoses in 23% of patients. Our predictive tool may guide clinicians in refining pretest probability and optimizing diagnosis for ATTR-CM. External validation in broader populations is warranted.

Cardiac amyloidosis is a common etiology of infiltrative cardiomyopathy characterized by deposition of insoluble protein fibrils, leading to heart failure (HF) and arrhythmias, especially among elderly patients. The most prevalent subtypes of cardiac amyloidosis include transthyretin amyloid cardiomyopathy (ATTR-CM), both wild-type and variant, and light chain amyloid (AL) cardiomyopathy. Autopsy studies have demonstrated transthyretin amyloid deposits in 19% of patients with a history of HF with preserved ejection fraction (HFpEF).^1^ One prospective study identified clinically significant ATTR-CM in 13% of 120 patients aged over 60 years hospitalized with HFpEF.^2^ ATTR-CM remains underdiagnosed, typically identified at advanced stages following numerous physician encounters.^3^

Small observational studies exploring differences between ATTR-CM vs non-ATTR-CM patients have shown that ATTR-CM patients are less likely to have hypertension, but more likely to present with increased left ventricular (LV) wall thickness, enlarged left atria, and severe diastolic dysfunction. ATTR-CM patients hospitalized with HFpEF also exhibit elevated median N-terminal pro–B-type natriuretic peptide (BNP) (NT-proBNP) and troponin I values, along with a higher prevalence of atrial fibrillation (AF) and pacemaker implantation.^2^ In elderly patients undergoing transcatheter aortic valve replacement (TAVR), ATTR-CM is associated with greater interventricular septal thickness (IVSD), increased LV mass index, lower stroke volume index, advanced diastolic dysfunction, and reduced LV systolic function compared to those without ATTR-CM.^4^

Recent advancements in noninvasive imaging and the advent of disease-modifying therapies have improved the rate of ATTR-CM diagnosis, underscoring the importance of timely and accurate diagnoses. Among high-risk patients, including those with HF, AF, and aortic stenosis (AS), the prevalence of ATTR-CM is increasingly recognized. However, the clinical heterogeneity in ATTR-CM presentation, coupled with variable familiarity with this diagnosis particularly among clinicians in the community continues to pose challenges for routine screening. Identification of high-risk clinical and imaging features may enhance efficient and accurate ATTR-CM diagnosis. Therefore, we conducted a prospective study within specialized HF, AF, and TAVR clinic to: 1) describe the prevalence of ATTR-CM in predefined high-risk populations referred for technetium-99m-pyrophosphate (^99m^Tc-PYP) imaging; 2) identify predictors of ATTR-CM; and 3) developed a novel predictive tool to facilitating early and accurate diagnosis of ATTR-CM in this populations.

## Methods

### Study design and selection of participants

We conducted a prospective observational registry study where patients were screened at dedicated HF, AF, and TAVR clinics across 2 academic hospitals in Vancouver, Canada, from January 2019 to December 2022. These high pretest probability specialty clinics functioned as structured screening pathways or funnels for ATTR-CM evaluation. Patients aged over 60 years referred for ^99m^Tc-PYP nuclear scintigraphy were approached for participation immediately after their cardiology visit and before undergoing their ^99m^Tc-PYP scan. The study was approved by the University of British Columbia Clinical Research Ethics Board (H18-03139).

Patients were eligible if they met one or more of the following inclusion criteria: 1) symptomatic HF with a) LVEF >40% or b) LVEF <40% and normal LV size (defined by American Society of Echocardiography Chamber Quantification Guidelines as normal LV end diastolic volume indexed to body surface area);^5^ 2) maximum LV wall thickness ≥12 mm; 3) moderate or severe diastolic dysfunction or restrictive filling pattern (defined by the American Society of Echocardiography 2016 Diastology Guidelines;^6^ 4) age >70 years old and new HF presentation; 5) low-flow low-gradient AS (aortic valve area ≤1 cm^2^ and mean aortic valve gradient <40 mm Hg or peak velocity <4 m/s); 6) severe AS with average mitral annular systolic velocity by tissue Doppler (S’) ≤6 mm/s; or 7) BNP or NT-proBNP > upper limit of normal (ULN) in NYHA functional class I, >2 × ULN in NYHA functional class II, and >2.5 × ULN in NYHA functional class III. The rationale for the selection of these inclusion criteria include previous small studies^2^^,^^7^, ^8^, ^9^, ^10^, ^11^ and our own clinical experience.

Patients were excluded if they had a previously confirmed diagnosis of AL or ATTR amyloidosis, history of endomyocardial biopsy negative for amyloid by Congo red staining, history of negative cardiac ^99m^Tc-PYP nuclear scintigraphy, genetic testing positive for any mutation associated with hypertrophic cardiomyopathy, abnormal alpha-galactosidase testing or positive genetic testing for Fabry disease, or HF caused by ischemic cardiomyopathy or valvular disease (except AS).

A new ATTR-CM diagnosis was defined as a positive ^99m^Tc-PYP in the absence of a monoclonal protein. Those with elevated light chains underwent further hematologic evaluation to exclude AL amyloidosis.

Baseline characteristics, cardiac imaging, and biochemical results were collected. Follow-up was conducted via phone or subsequent clinic visits.

### Statistical analyses

Missing data at baseline were infrequent (<1% for most variables). Missing values were imputed using multiple imputations under the assumption “missing at random” and the number of imputations to be performed was specified as 5 for higher accuracy. All analyses were conducted with complete data. Baseline characteristics of study participants in each cohort were reported using mean (±SD) or median with IQR, as appropriate, for continuous variables, and frequencies (proportions) for categorical variables. Baseline characteristics and outcomes were compared between patients with confirmed clinical diagnosis of ATTR-CM and non ATTR-CM patients, using a two-sample Student’s t-test, Wilcoxon rank-sum test, Fisher exact test, and chi-square test, as appropriate.

Using a logistic regression model, an ATTR-CM prediction model was developed to identify patients at high risk for having ATTR-CM. Candidate variable selection was based previously well-documented features of ATTR-CM.^12^ Continuous variables were transformed to categorical variables using predetermined cutoffs or median values to facilitate the creation of a practical risk score. Associations between ATTR-CM and candidate variables in our cohort were analyzed using univariate logistic regression. Risk factors with a *P* value of <0.20 in the univariable analysis were then considered in the initial multivariable logistic model. Collinearity between variables was assessed using the variance inflation factor. A model selection procedure using Least Absolute Shrinkage and Selection Operator regression and based on the Akaike information criterion was used to select risk factors in the final model. Model performance was evaluated by assessing both discrimination and calibration. Discrimination, which refers to the model’s ability to distinguish between patients with and without ATTR-CM, was assessed using the area under receiver operating characteristic curve (AUROC). Calibration, which refers to the agreement between observed outcomes and model predictions, was evaluated using graphical methods and the Hosmer-Lemeshow test. Furthermore, internal validation of the model was performed using a bootstrapping technique to account for model over-fitting.^13^ Two hundred bootstrapping samples were created with 500 replications. The final predictive diagnostic algorithm to identify patients with ATTR-CM was developed using the independent predictive variables in the final model. The optimal score for cutoff (0.22) was determined by balancing sensitivity and specificity (maximizing Youden index), favoring sensitivity. As this is a predictive tool, false positives would lead only to additional testing, but false negatives would result in a missed diagnoses and potentially worse outcomes. Finally, to simplify the model into a tool that can be applied in clinical practice, we developed a simplified weighted risk score using variable ß coefficients and confirmed that our risk score cutoff for further testing had the same test characteristics as the complex model.

The conventional *P* < 0.05 level of significance was used as a nominal reporting level, and all reported *P* values were 2 tailed. All statistical analyses were performed using SAS software (version 9.4; SAS Institute) and R software (version 4.4.1; R Foundation for Statistical Computing).

## Results

### Prevalence and characteristics of the patient population with ATTR-CM

**Figure 1:**
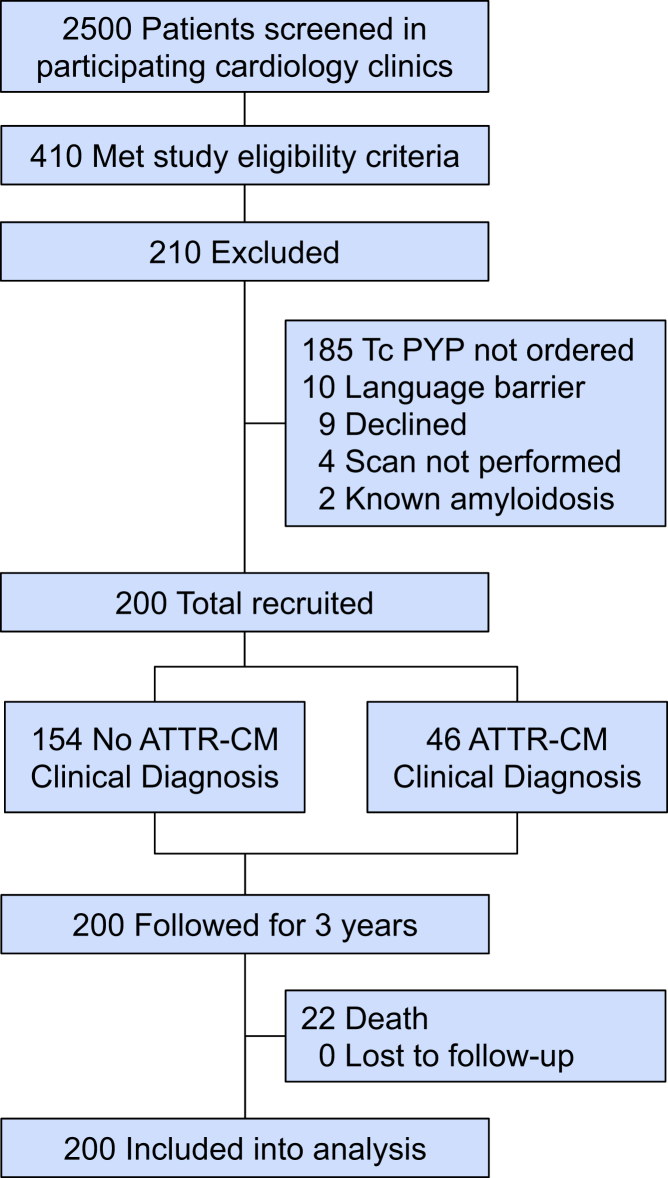
**Study Cohort and Exclusions** Flow diagram of patients screened across high pretest probability clinics (heart failure, atrial fibrillation, and TAVR) and referred for ^99m^Tc-PYP imaging. The figure outlines initial screening numbers, exclusions, completion of diagnostic testing, and final cohort included in model derivation. ATTR-CM = transthyretin amyloid cardiomyopathy; Tc PYP = technetium pyrophosphate.

In our study, 2,500 patients were screened and 410 met the study eligibility criteria (Figure 1). Of these patients, 210 were excluded, including 185 who did not have ^99m^Tc-PYP nuclear scintigraphy performed, due to cardiologist discretion. In total, 200 patients were enrolled in this study (Central Illustration). Table 1 describes the baseline characteristics of the study population. The mean age was 78.4 ± 8.3 years, 64.5% were males, and 72.0% of patients were Caucasian. History of HF was seen in 83.0% of all patients with a mean duration of 0.86 years from onset of HF to time of ATTR-CM diagnosis.

**Central Illustration:**
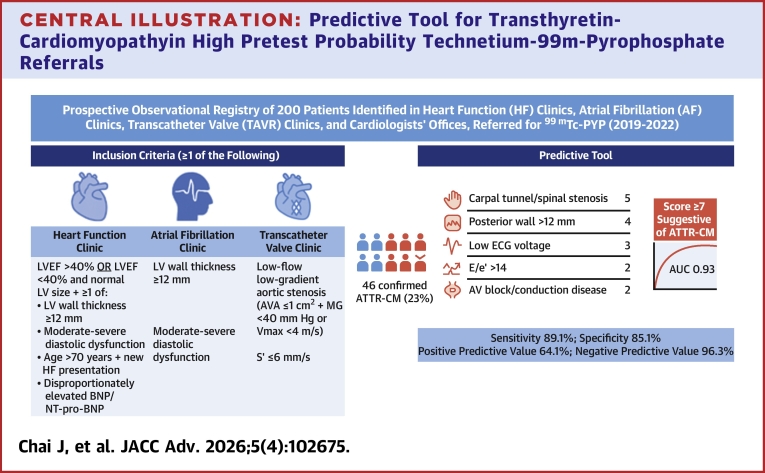
**Predictive Tool for Transthyretin-Cardiomyopathyin High Pretest Probability Technetium-99m-Pyrophosphate Referrals** Overview of the predictive tool developed for identifying ATTR-CM in high pretest probability patients referred for ^99m^Tc-PYP imaging. The illustration summarizes key clinical, electrocardiographic, and echocardiographic predictors incorporated into the final model and weighted score. ^99m^Tc-PYP = technetium-99m-pyrophosphate; ATTR-CM = transthyretin-cardiomyopathy; AUC = area under the curve; AVA = aortic valve area; BNP = brain natriuretic peptide; ECG = electrocardiogram; E/e’ = ratio of early mitral inflow velocity to mitral annular early diastolic velocity; LV = left ventricular; LVEF = left ventricular ejection fraction; LVPWD = left ventricular posterior wall diameter; NT-proBNP = N-terminal prohormone brain natriuretic peptide; S’ = right ventricular systolic excursion velocity.

**Table 1:** Baseline Characteristics Stratified by ATTR-CM and No ATTR-CM Diagnosis

	Overall (N = 200)	No ATTR-CM Clinical Diagnosis (n = 154)	ATTR-CM Clinical Diagnosis (n = 46)	*P* Value
Age (years)	78.4 ± 8.3	77.2 ± 8.1	82.6 ± 7.1	<0.001
>70 years	168 (84.0)	123 (79.9)	45 (97.8)	0.002
Male	129 (64.5)	92 (59.7)	37 (80.4)	0.013
Ethnic background				
Caucasian	144 (72.0)	108 (70.1)	36 (78.3)	
East Asian	38 (19.0)	32 (20.8)	6 (13.0)	
South Asian	9 (4.5)	8 (5.2)	1 (2.2)	
Hispanic or Latino	1 (0.5)	1 (0.6)	0 (0)	
African Canadian	3 (1.5)	2 (1.3)	1 (2.2)	
Other	5 (2.5)	3 (1.9)	2 (4.3)	
History of diabetes	53 (26.5)	43 (27.9)	10 (21.7)	0.452
History of hypertension	158 (79.0)	124 (80.5)	34 (73.9)	0.409
History of dyslipidemia	115 (57.8)	88 (57.5)	27 (58.7)	1.000
Previous or current smoker	69 (34.5)	47 (30.5)	22 (48.9)	0.028
History of coronary artery disease	59 (29.5)	44 (28.6)	15 (32.6)	0.586
History of myocardial infarction	26 (13.0)	23 (14.9)	3 (6.5)	0.210
History of HF	166 (83.0)	121 (78.6)	45 (97.8)	0.001
Time from HF onset (years)	0.86 (0.2, 2.3)	0.83 (0.3, 2.2)	0.90 (0.2, 2.8)	0.759
Hospitalization for HF in past year	71 (42.8)	58 (47.9)	13 (28.9)	0.034
History of valve surgery or TAVR	11 (5.5)	8 (5.2)	3 (6.5)	0.718
Atrial fibrillation or atrial flutter	116 (58.0)	87 (56.5)	29 (63.0)	0.497
Pacemaker or ICD	40 (20.0)	28 (18.2)	12 (26.1)	0.293
History of CVA	41 (20.5)	30 (19.5)	11 (23.9)	0.535
Moderate or severe AS or prosthetic valve	40 (20)	32 (20.8)	8 (17.4)	0.680
Carpal tunnel or trigger finger	54 (27.0)	22 (14.3)	32 (69.6)	<0.001
Spinal stenosis (any segment)	40 (20.0)	14 (9.1)	26 (56.5)	<0.001
NYHA functional class				0.001
I	26 (13.0)	23 (14.9)	34 (6.5)	
II	123 (61.5)	101 (65.6)	22 (47.8)	
III	49 (24.5)	30 (19.5)	19 (41.3)	
IV	2 (1.0)	0 (0)	2 (4.3)	
Low voltage ECG (limb leads)	23 (11.5)	6 (3.9)	17 (37.0)	<0.001
Voltage to LV mass ratio	0.17 ± 0.1	0.18 ± 0.1	0.13 ± 0.1	0.003
Pseudoinfarct on ECG^a^	22/149 (14.8)	7/120 (5.8)	15/29 (51.7)	<0.001
Any atrioventricular block (AVB)^a^	43/185 (23.2)	28/146 (19.2)	15/39 (38.5)	0.018
Any intraventricular conduction delay^a^	65/185 (35.1)	46/146 (31.5)	22/39 (48.7)	0.045
Glomerular filtration rate	53.9 ± 20.0	54.0 ± 20.4	53.3 ± 18.3	0.852
Troponin any elevation	92 (57.5)	55 (47.4)	37 (84.1)	<0.001
Troponin >2x elevation	67 (41.9)	40 (34.5)	27 (61.4)	0.004
NT proBNP level (ng/L)	2,256 (988, 4,730)	2018 (922, 3,789)	3,633 (2,193, 5,766)	0.027
BNP any elevation	157 (86.3)	114 (83.2)	43 (95.6)	0.045
BNP >5x elevation	91 (50.0)	62 (45.3)	29 (64.4)	0.039
Echocardiogram parameters				
Moderate or severe diastolic dysfunction^a^	69/105 (65.7)	46/75 (61.3)	23/30 (76.7)	0.174
IVSD (mm)	12.7 ± 2.9	11.9 ± 2.1	15.3 ± 3.4	<0.001
LVPWD (mm)	11.9 ± 2.6	11.2 ± 1.8	14.5 ± 3.2	<0.001
LVEDD (mm)	47.4 ± 8.3	48.6 ± 8.0	43.3 ± 7.7	<0.001
LVEF (%)	55.0 (45, 61)	55.0 (43, 60)	56.5 (49, 61)	0.617
E/e’	15.2 ± 6.8	14.4 ± 6.2	18.7 ± 7.6	0.001
GLS (%) n = 42	−12.2 ± 3.6	−11.9 ± 3.7	−12.4 ± 3.4	0.644
Baseline cardiac medications				
Beta-blocker	119 (59.5)	104 (67.5)	15 (32.6)	<0.001
ACE inhibitors or ARB	99 (49.5)	84 (54.5)	15 (32.6)	0.011
Sacubitril/valsartan	16 (8.0)	13 (8.4)	3 (6.5)	1.000
Calcium channel blocker	58 (29.0)	50 (32.5)	8 (17.4)	0.063
Diuretic	133 (66.5)	97 (63.0)	36 (78.3)	0.074
Digoxin	8 (4.0)	8 (5.2)	0 (0)	0.202
Mineralocorticoid antagonist	75 (37.5)	55 (35.7)	20 (43.5)	0.387
Amiodarone	18 (9.0)	9 (5.8)	9 (19.6)	0.008
Oral anticoagulant	100 (50.0)	76 (49.4)	24 (52.2)	0.867
SGLT2 inhibitor	21 (10.5)	20 (13.0)	1 (2.2)	0.051

Values are n (%), median (Q1, Q3), or mean ± SD.

*P* value represents the difference between ATTR-CM diagnosis groups.

ACE = angiotensin-converting-enzyme inhibitor; ARB = angiotensinogen receptor blocker; AS = aortic stenosis. ATTR-CM = transthyretin amyloid cardiomyopathy; BNP = B-type natriuretic peptide; CVA = cerebrovascular accident; ECG = electrocardiogram; E/e’ = ratio of early mitral inflow velocity to mitral annular early diastolic velocity; GLS = global longitudinal strain; HF = heart failure; ICD = implantable cardioverter-defibrillator; IVSD = intraventricular septal thickness; LV = left ventricular; LVEDD = left ventricular end diastolic diameter; LVEF = left ventricular ejection fraction; LVPWD = left ventricular posterior wall thickness; NT-proBNP = N-terminal pro–B-type natriuretic peptide; SGLT2 = sodium-glucose cotransporter 2; TAVR = transcatheter aortic valve replacement.

a Not used in multiple imputation, calculation based on complete data.

Of the 200 patients enrolled, 46 (23%) received a diagnosis of ATTR-CM. Compared with non–ATTR-CM, ATTR-CM patients tended to be older (82.6 ± 7.1 years vs 77.2 ± 8.1 years, *P* < 0.001) and a larger proportion were males (80.4 vs 59.7%, *P* = 0.013). They were more likely to report NYHA functional class III/IV symptoms (45.6% vs 19.5%, *P* = 0.001) and had higher NT-proBNP levels (3,633 [2,193, 5,766] vs 2018 [922, 3,789]; *P* = 0.027). More ATTR-CM patients had a history of carpal tunnel syndrome (69.6% vs 14.3%, *P* < 0.001) and spinal stenosis (56.5% vs 9.1%, *P* < 0.001). There were no differences in other baseline comorbidities including history of diabetes, hypertension, dyslipidemia, AF, moderate or severe AS or prosthetic valve, pacemaker or implantable cardioverter-defibrillator insertion, transient ischemic attack or stroke, coronary artery disease, or previous myocardial infarction. There were also no differences in history of autonomic dysfunction or peripheral neuropathy. Among patients with ATTR-CM, 63.0% had AF, and none had AF without HF. Baseline use of most cardiac medications was similar between ATTR-CM vs non–ATTR-CM groups with less frequent use of beta-blockers (32.6% vs 67.5%, *P* < 0.001) and renin angiotensin antagonists (32.6% vs 54.5%, *P* = 0.011), and more frequent use of amiodarone (19.6% vs 5.8%, *P* = 0.008) observed in the ATTR-CM group. There was a trend to more frequent use of diuretic agents (78.3% vs 63.0%, *P* = 0.074) in the ATTR-CM patients.

### Clinical and imaging characteristics predictive of ATTR-CM

More ATTR-CM patients than non–ATTR-CM had findings of low voltage QRS complexes defined as QRS amplitude <5 mm in all limb leads (37.0% vs 3.9%, *P* < 0.001), low voltage to LV mass index ratio (0.13 ± 0.1 vs 0.18 ± 0.1, *P* = 0.003), pseudoinfarct pattern (51.7% vs 5.8%, *P* < 0.001), or any atrioventricular (AV) block (38.5% vs 19.2%, *P* = 0.018) on electrocardiogram (ECG).

Echocardiographic findings differed significantly among patients. Compared to non–ATTR-CM, ATTR-CM patients had larger IVSD (15.3 ± 3.4 vs 11.9 ± 2.1; *P* < 0.001) and larger LV posterior wall thickness (LVPWD) (14.5 ± 3.2 vs 11.2 ± 1.8; *P* < 0.001). ATTR-CM patients had smaller LV end-diastolic diameter (43.3 ± 7.7 vs 48.6 ± 8.0, *P* < 0.001). E/e’ was higher in ATTR-CM patients (18.7 ± 7.6 vs 14.4 ± 6.2; *P* = 0.001). There were no significant differences in ejection fraction, left atrial volume index, or global longitudinal strain, although for the latter variable, data were only available for 21% of patients. Of those with ATTR-CM, 98% were >70 years of age or had a history of HF, and only 6% had dilated left ventricles.

### Sensitivity, specificity, positive, and negative predictive values of clinic screening criteria in patients undergoing ^99m^Tc-PYP scan

The positive predictive value (PPV) for ATTR-CM screening criteria ranged from 16 to 38% (Table 2): highest for age >70 years and new HF (PPV 38%), moderate-severe diastolic dysfunction (PPV 34%), and IVSD ≥12 mm (PPV 32%). The negative predictive value (NPV) was highest for age >70 and new onset HF (NPV 95%), followed by HFpEF and IVSD (NPV 89% for each).

**Table 2:** Inclusion Criteria Test Characteristics for ATTR-CM

	Sensitivity (%)	Specificity (%)	PPV (%)	NPV (%)
HFpEF	43/50 (86)	54/150 (36)	43/139 (31)	54/61 (89)
HFrEF with normal LVEDD	6/50 (12)	126/150 (84)	6/30 (20)	126/170 (74)
IVSD ≥12 mm	43/50 (86)	58/150 (39)	43/135 (32)	58/65 (89)
Moderate- severe diastolic dysfunction	27/50 (54)	98/150 (65)	27/79 (34)	98/121 (81)
Age >70 y and new onset HF	46/50 (92)	74/150 (49)	46/122 (38)	74/78 (95)
Low-flow low-gradient severe AS	3/50 (6)	134/150 (89)	3/19 (16)	134/181 (74)
Disproportionally elevated BNP	38/50 (76)	48/150 (32)	38/140 (27)	48/60 (80)

HFpEF = heart failure with preserved ejection fraction; HFrEF = heart failure with reduced ejection fraction; NPV = negative predictive value; PPV = positive predictive value; other abbreviations as in Table 1.

### Final diagnoses of patients undergoing ^99m^Tc-PYP scan and outcomes of patients with ATTR-CM

**Figure 2:**
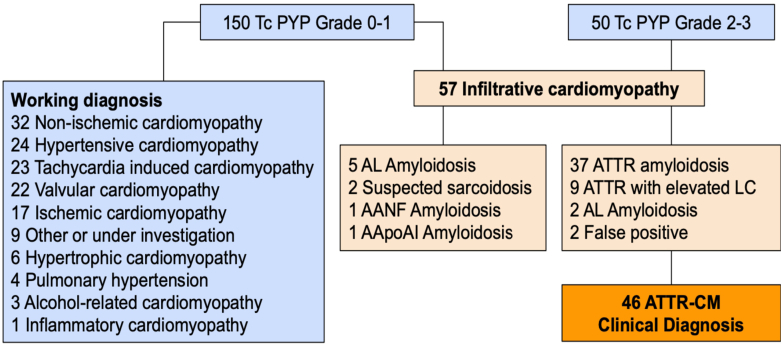
**Final Diagnoses of Screened Patient Stratified by Technetium-99m-Pyrophosphate Results** Distribution of final diagnoses stratified by ^99m^Tc-PYP imaging results. Patients with positive scans underwent confirmatory evaluation to distinguish ATTR-CM from other causes of myocardial uptake. AANF = atrial natriuretic factor amyloidosis; AApoA1 = apolipoprotein A1 amyloidosis; AL = light chain amyloid; LC = light chains; other abbreviations as in Figure 1.

Figure 2 summarizes the final diagnoses of screened patients. A total of 46 patients had a final diagnosis of ATTR-CM, of whom 9 patients had elevated light chains. Seven (3.5%) patients were diagnosed with AL amyloidosis including 2 patients who had a positive ^99m^Tc-PYP scan. Two patients had a false positive ^99m^Tc-PYP scan test, with subsequent negative endomyocardial biopsies. Of those that had a negative ^99m^Tc-PYP scan, 32 (21.3%) of patients with nonischemic cardiomyopathy, 24 (16.0%) with hypertensive cardiomyopathy, 23 (15.3%) with tachycardia-induced cardiomyopathy, and 22 (14.7%) with valvular cardiomyopathy. Eleven patients (5.5%) had a cardiac biopsy revealing 6 confirmed ATTR-CM, 3 AL amyloidosis, and 2 normal biopsy results.

### Predictive diagnostic algorithm identifying patients with ATTR-CM

After univariate analysis, thirteen candidate variables (Table 3) met the criteria for clinical relevance and *P* < 0.20. These included age, male sex, history of HFpEF, history of either carpal tunnel syndrome or lumbar spinal stenosis, any AV block or interventricular conduction delay, low QRS voltage on ECG, disproportional elevation in BNP or NT-proBNP (defined as > ULN in NYHA functional class I; >2 times the ULN in NYHA functional class II; more than 2.5 times the ULN in NYHA functional class III and IV), elevated troponin, E/e’ >14, IVSD >12 mm, LVPWD >12 mm, maximum wall thickness >12 mm, and relative wall thickness >0.49. There was significant colinearity between IVSD, LVPWD, maximum wall thickness, and relative wall thickness. LVPWD had the strongest association with ATTR-CM and was retained in the list of candidate variables whereas the others were eliminated. The following 5 predictors of ATTR-CM were included in our final model including: 1) history of either of carpal tunnel syndrome or lumbar spinal stenosis; 2) AV block or intraventricular conduction delay on ECG; 3) low QRS complex voltages on ECG; 4) diastolic dysfunction with E/e’>14; and 5) LVPWD >12 mm (Table 4). Our model showed good discrimination ability with an AUROC of 0.931 (0.884-0.978) (Supplemental Figure 1). The model continued to have good discrimination ability during internal validation: the optimism-corrected AUROC was 0.921 for bootstrapped samples and the estimated optimism was 0.010. The calibration plot of the model (Supplemental Figure 2) showed fair calibration and the calibration was also reasonable when assessed by the Hosmer and Lemeshow Goodness-of-Fit test (*P* = 0.35) (Supplemental Figure 2). Accordingly, the final model for prediction was risk factor score (for ATTR-CM) = 1/(1+exp(-A)), where

**Table 3:** Univariate Logistic Regression Analysis

	OR	Lower 95% CI	Upper 95% CI	*P* Value
Age (y)	1.093	1.042	1.146	<0.001
Sex	2.667	1.216	5.852	0.014
HFpEF	3.476	1.417	8.528	0.007
Carpal tunnel syndrome or lumbar spinal stenosis	16.79	7.387	38.18	<0.001
Any AV block or IVCD	3.956	1.851	8.453	<0.001
Low voltage ECG	13.55	4.979	36.89	<0.001
BNP or NT-proBNP elevation	3.183	0.806	12.56	0.100
Troponin elevation	3.559	1.522	8.324	0.003
E/e’> 14	4.626	2.163	9.896	<0.001
IVSD >12 mm	3.883	1.586	9.506	0.003
LVPWD >12 mm	9.729	4.609	20.54	<0.001
Maximum LV wall thickness >12 mm	7.277	3.308	16.01	<0.001
Relative wall thickness >0.49	8.688	3.714	20.32	<0.001

AV = atrioventricular; IVCD = intraventricular conduction delay; other abbreviations as in Tables 1 and 2.

**Table 4:** Analysis of Penalized Maximum Likelihood Estimates (LASSO Regression, Tuning Parameter = 0.0366)

	Estimate	SE	95% CI	*P* Value
Intercept	−4.730	0.653	−0.610 to −3.449	<0.001
Carpal tunnel syndrome or lumbar spinal stenosis	2.430	0.506	1.437-3.442	<0.001
LVPWD >12 mm	1.813	0.499	0.834-2.791	<0.001
Low voltage ECG	1.963	0.713	0.566-3.359	0.006
E/e’ > 14	1.127	0.524	0.099-2.154	0.032
Any AV block or IVCD	1.045	0.512	0.040-2.049	0.042

Hosmer and Lemeshow Goodness-of-Fit *P* = 0.35, AUROC = 0.931 (0.884-0.978), AIC = 108.4 and McFadden's R-Square = 0.51.

LASSO = Least Absolute Shrinkage and Selection Operator; other abbreviations as in Tables 1 and 3.

A = −4.730 + 2.430∗Carpal tunnel and lumbar stenosis +1.813∗LVPWD >12 mm + 1.963∗Low voltage ECG +1.127∗E/e’ >14 + 1.045∗Any AV block or IVCD.

Using a cutoff of 0.22 for this model, we found a sensitivity of 89.1% (80.1%-98.1%), specificity of 85.1% (70.4%-90.7%), PPV of 64.1% (52.3%-75.8%), and NPV of 96.3% (93.2%-99.5%). An example illustrated how to predict the risk score using the previously mentioned final model was shown in Supplemental Material.

**Figure 3:**
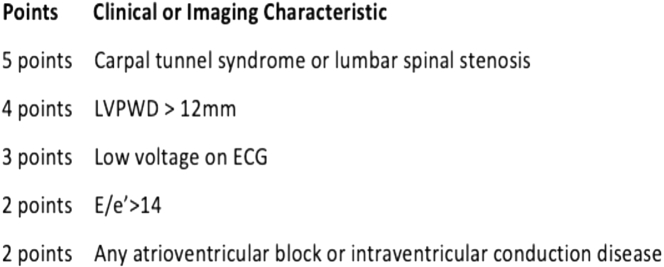
**Prediction Tool for Transthyretin-Cardiomyopathy** Graphical representation of the multivariable prediction model identifying independent predictors of ATTR-CM in high pretest probability patients referred for ^99m^Tc-PYP imaging. ECG = electrocardiogram; LVPWD = left ventricular posterior wall thickness.

To simplify the model into a tool that can be applied in clinical practice, we developed a simplified risk score (Figure 3). We used the beta coefficients of the final model to assign the points. The assignment was referred to the value of linear predictor A related to the cutoff point (probability = 0.22) which equals to −1.266 (the minimum value for identification). For example, the sum of the biggest and the smallest beta coefficients (2.430, 1.045, respectively) and the intercept (−4.730) equals to −1.255 >-1.266, accordingly, the biggest weight of score was arranged for “carpal tunnel or spinal stenosis” (point score = 5), and the smallest weight of score was arranged for “any AV block or intraventricular conduction disease” (point score = 2). The remaining point scores were based on the differences relative to −1.255.

We performed a nonlinear regression model to test the association of estimated point score from the final model and the simplified risk score using our sample data which was expressed as: simplified risk score = 0.744 + 45.959∗(estimated point score) −84.028∗(estimated point score)^2^ + 54.622∗(estimated point score)^3^ (adjusted R-square = 0.98, F-test *P* < 0.001, residuals scattered randomly around 0). For example, if estimated point score = 0.22, then simplified risk score ≈7.37. This strong association validated that by using a cutoff of 7 for further testing, it had the same test characteristics as the estimated point score from the complex model. This cutoff was determined balancing sensitivity and specificity, as outlined previously.

## Discussion

We identified clinical and imaging characteristics to predict a diagnosis of ATTR-CM in 200 high-risk patients followed in clinics specializing in HF, AF, and TAVR, referred for ^99m^Tc-PYP where 46 patients had a final diagnosis of ATTR. We created our own pragmatic, predictive model to identify ATTR-CM in these high-risk patient clinic populations referred for ^99m^Tc-PYP. Our most significant findings include: 1) our predictive model has good performance characteristics with sensitivity of 89.1% and specificity of 85.1% to predict ATTR-CM, 2) among the 5 characteristics, the presence of carpal tunnel or spinal stenosis had the highest predictive value for ATTR-CM, followed by echocardiographic and ECG findings; 3) screening criteria test characteristics for this study had the highest sensitivity and NPVs for age >70 and new onset HF, then HFpEF, and then LV wall thickness ≥12 mm. This is the first prospective study to develop a diagnostic screening algorithm for the identification of ATTR-CM patients among diverse specialized clinic populations at high risk of ATTR-CM.

Previous studies have retrospectively evaluated cohorts to determine predictive criteria of diagnosing ATTR-CM via ^99m^Tc-PYP^6^^,^^7^ or endomyocardial biopsies.^8^ One study evaluated patients with the possibility of ATTR-CM (defined as cardiac symptoms or cardiovascular disorders (not specified) with at least 1 other clinical manifestation) who underwent ^99m^Tc-PYP and validated the “Kumamoto criteria” which assigned 1 point for each of: 1) high sensitivity troponin ≥0.0308 ng/mL; 2) LVPWD ≥13.6 mm; and 3) QRS ≥120 ms with 3 points correlating to 89% positivity for ^99m^Tc-PYP.^14^ Another similar study^15^ evaluated the “T-Amylo” score which used carpal tunnel, age, male, IVSD, and low QRS voltage. This study preidentified patients undergoing ^99m^Tc-PYP scans with IVSD ≥12 mm with 1 red flag feature. Their prediction tool was then validated in 11 centers. Investigators at the Mayo clinic also developed a simple scoring tool compromising age, sex, hypertension, ejection fraction, LVPWD, and relative wall thickness. They used a retrospective cohort to predict risk of ATTR-CM in a specific subset of patients with HFpEF that underwent ^99m^Tc-PYP. Another study assessed patients with HFpEF with ejection fraction ≥50% referred for endomyocardial biopsy (due to HF with unexplained restrictive features or unexplained LV hypertrophy) and found multivariable predictors of ATTR-CM included: 1) age >50 years; 2) peripheral neuropathy; and 3) IVSD ≥14 mm.^16^

In contrast to these previous predictive models, our study differs in the following ways: 1) this was a prospective study; and 2) we identified a cohort of high-risk patients in HF, AF, and TAVR clinics referred for ^99m^Tc-PYP scans, creating a more practical tool for specialty outpatient clinics with enriched patient populations. The Mayo score is limited to a HFpEF population, and the T-Amylo score is limited to those meeting IVSD ≥12 mm and 1 of 15 red flags or clinical scenarios defined by the European Society 2021 Position Paper, which may be more complex to implement into outpatient clinics. Similar to the T-Amylo score, our prediction tool also takes into account clinical syndromes (carpal tunnel), as well as the addition of spinal stenosis, ECG, and echocardiographic variables.

With the advent of new treatments for ATTR-CM, it is imperative that the disease is diagnosed early, at a point when patients are most likely to respond to therapy. We propose the application of our predictive tool specifically in high-risk clinic populations referred for ^99m^Tc-PYP meeting our inclusion criteria. Next steps including validation of our predictive model in other patient populations.

## Study Limitations

First, this study included a relatively small number of patients at a single center. Second, diagnoses of ATTR-CM in our cohort were largely based on ^99m^Tc-PYP scans and few patients underwent endomyocardial biopsy. Thirdly, because referral for ^99m^Tc-PYP was at the discretion of the clinic cardiologist, some patients who may have met our predefined high-risk inclusion criteria might not have been referred and therefore were not captured in this registry. This referral-based inclusion likely enriched our cohort with patients who had a higher pretest probability of ATTR-CM, which may limit generalizability to broader, unselected clinic populations. Finally, our tool was developed and validated in a high-risk cohort referred for ^99m^Tc-PYP with relatively advanced disease, which may limit its applicability to lower-risk populations with earlier-stage disease, in whom early identification and treatment have been shown to yield greater benefit.

## Conclusions

Broad screening criteria applied to high-risk patient populations referred for ^99m^Tc-PYP scintigraphy yielded new ATTR-CM diagnoses in 23% of patients. We developed a predictive model for ATTR-CM using a combination of carpal tunnel or spinal stenosis, LVPWD, low voltage on ECG, E/e’, and conduction disease in a high-risk clinic patient population. This tool may assist clinicians in identifying patients from diverse high-risk clinic populations and guide clinicians in refining pretest probability and diagnostic evaluation for suspected ATTR-CM. Further studies are needed to validate our predictive model amongst broader populations.

## Funding support and author disclosures

This study was funded by 10.13039/100004319Pfizer (grant-in-aid). The authors have reported that they have no relationships relevant to the contents of this paper to disclose.

## Supplementary Material

Supplementary docx 1

## References

[1] Mohammed S.F., Mirzoyev S.A., Edwards W.D. (2014). Left ventricular amyloid deposition in patients with heart failure and preserved ejection fraction. JACC Heart Fail.

[2] González-López E., Gallego-Delgado M., Guzzo-Merello G. (2015). Wild-type transthyretin amyloidosis as a cause of heart failure with preserved ejection fraction. Eur Heart J.

[3] Lousada I., Comenzo R.L., Landau H., Guthrie S., Merlini G. (2015). Light chain amyloidosis: patient experience survey from the amyloidosis research consortium. Adv Ther.

[4] Castano A., Narotsky D.L., Hamid N. (2017). Unveiling transthyretin cardiac amyloidosis and its predictors among elderly patients with severe aortic stenosis undergoing transcatheter aortic valve replacement. Eur Heart J.

[5] Lang R.M., Badano L.P., Mor-Avi V. (2015). Recommendations for cardiac chamber quantification by echocardiography in adults: an update from the American Society of Echocardiography and the European Association of Cardiovascular Imaging. J Am Soc Echocardiogr.

[6] Nagueh S.F., Smiseth O.A., Appleton C.P. (2016). Recommendations for the evaluation of left ventricular diastolic function by echocardiography: an update from the American Society of Echocardiography and the European Association of Cardiovascular Imaging. J Am Soc Echocardiogr.

[7] Barbhaiya C.R., Kumar S., Baldinger S.H. (2016). Electrophysiologic assessment of conduction abnormalities and atrial arrhythmias associated with amyloid cardiomyopathy. Heart Rhythm.

[8] Magdi M., Mostafa M.R., Abusnina W. (2022). A systematic review and meta-analysis of the prevalence of transthyretin amyloidosis in heart failure with preserved ejection fraction. Am J Cardiovasc Dis.

[9] Abouezzeddine O.F., Davies D.R., Scott C.G. (2021). Prevalence of Transthyretin amyloid Cardiomyopathy in heart failure with preserved ejection fraction. JAMA Cardiol.

[10] Rosenblum H., Masri A., Narotsky D.L. (2021). Unveiling outcomes in coexisting severe aortic stenosis and transthyretin cardiac amyloidosis. Eur J Heart Fail.

[11] Aimo A., Camerini L., Fabiani I. (2023). Valvular heart disease in patients with cardiac amyloidosis. Heart Fail Rev.

[12] Maurer M.S., Bokhari S., Damy T. (2019). Expert consensus recommendations for the suspicion and diagnosis of transthyretin cardiac amyloidosis. Circ Heart Fail.

[13] Lankham I., Slaugther M. (2020). Simple and efficient bootstrap validation of predictive models using SAS/STAT® software. SAS Glob Forum.

[14] Ochi Y., Kubo T., Baba Y. (2021). Validation of the Kumamoto criteria for prediction of 99m technetium pyrophosphate scintigraphy positivity as a strategy for diagnosis of transthyretin cardiac amyloidosis: a retrospective cohort study in Kochi. J Cardiol.

[15] Arana-Achaga X., Goena-Vives C., Villanueva-Benito I. (2023). Development and validation of a prediction model and score for transthyretin cardiac amyloidosis diagnosis: T-Amylo. JACC Cardiovasc Imaging.

[16] Ton V.K., Bhonsale A., Gilotra N.A. (2017). Baseline characteristics predict the presence of amyloid on endomyocardial biopsy. J Card Fail.

