# Supplementary material for: Development of a Predictive Tool in Patients With High Pretest Probability for Transthyretin Amyloid Cardiomyopathy
Source: JACC Adv. 2026 Mar 25;5(4):102675. doi: 10.1016/j.jacadv.2026.102675 (PMC13050072; doi:10.1016/j.jacadv.2026.102675)
Supplement: Supplementary docx 1 [file mmc1.docx]

**Supplementary**

**Figure S1. ROC curve.** Receiver operating characteristic (ROC) curve demonstrating discrimination of the multivariable model for identifying ATTR-CM in high pre-test probability patients referred for 99mTc-PYP imaging. The area under the curve (AUC) quantifies model performance in distinguishing patients with and without confirmed ATTR-CM. Confidence intervals for the AUC are shown.

**Figure S2. Predicted probability against observed proportion.** Calibration plot comparing predicted probabilities from the multivariable model with observed proportions of ATTR-CM across risk strata. Agreement between predicted and observed event rates demonstrates model calibration and reliability across increasing levels of predicted risk.

**An example illustrated how to predict the risk score using the final model**

| Individuals | Carpal tunnel and lumbar stenosis  (Yes=1, No=0) | LVPWD >12mm  (Yes=1, No=0) | Low voltage ECG  (Yes=1, No=0) | E/e’> 14  (Yes=1, No=0) | Any AV block or IVCD  (Yes=1, No=0) |
| --- | --- | --- | --- | --- | --- |
| Patient 1 | Yes | No | No | Yes | No |
| Patient 2 | No | Yes | Yes | No | Yes |
| Patient 3 | No | No | No | Yes | Yes |

Patient 1: A= -4.730 +2.430×1 +1.813×0 +1.963×0 +1.127×1 +1.045×0=-1.173

Risk score for (for ATTR-CM) = 1/(1+exp(-A)) =1/(1+exp(1.173) ≈ 0.24

Patient 2: A= -4.730 +2.430×0 +1.813×1 +1.963×1 +1.127×0 +1.045×1=0.091

Risk score for (for ATTR-CM) = 1/(1+exp(-A)) =1/(1+exp(-0.091) ≈ 0.52

Patient 3: A= -4.730 +2.430×0 +1.813×0 +1.963×0 +1.127×1 +1.045×1=-2.558

Risk score for (for ATTR-CM) = 1/(1+exp(-A)) =1/(1+exp(2.558) ≈ 0.07

Comments: patients 1 and 2 have risk score >0.22 (cutoff point) who would be identified as positive ATTR-CM; however, patient 3 has a risk score <0.22 who would be identified as negative ATTR-CM. exp: abbreviation for the exponential function.
